# Efforts to Improve the Seasonal Influenza Vaccine

**DOI:** 10.3390/vaccines8040645

**Published:** 2020-11-03

**Authors:** Rossella Cianci, Estelle E. Newton, Danilo Pagliari

**Affiliations:** 1 General Medicine, Fondazione Policlinico Universitario “Agostino Gemelli”, IRCCS, Università Cattolica del Sacro Cuore, 00168 Rome, Italy; danilo.pagliari@gmail.com; 2 CytoCure LLC, Beverly, MA 01915, USA; estelle.newton@gmail.com; 3 Medical Officer of the Carabinieri Corps, Carabinieri Officers School, 00165 Rome, Italy

Seasonal influenza is an acute syndrome, principally involving the respiratory tract caused by influenza viruses that are globally present. Every year, influenza is responsible for an estimated 3–5 million infections worldwide and about 290.000–650.000 total deaths [1]. Influenza may present different clinical features and courses causing high-rates of hospitalization particularly in frail patients with several comorbidities.

Influenza viruses belong to the *Orthomyxoviridae* family and may be divided into 3 principal types, Influenza A virus (IAV), Influenza B virus (IBV), and Influenza C virus (ICV). IAV and IBV are responsible for the vast majority of infections. 

There are several anti-viral drugs against influenza that act as neuroaminidase (NA) inhibitors, like oseltamivir, zanamivir, peramivir, and laninamivir that block virus reproduction and have been demonstrated effective in reducing severe cases and deaths [2,3,4].

However, the best practice to prevent influenza spreading remains the vaccination programs. To date, vaccination protocols are especially valuable in protecting elderly people (>65 years), frail patients with multiple comorbidities, cancer patients, people with chronic illnesses or weakened immune systems, pregnant women and health-care workers. Since statistics have revealed that the number of elderly people is continuously increasing and influenza mortality rates are higher in elderly people, more severe courses and more complications of influenza infection may be expected in the future. This fact further increases the need for an effective vaccination protocol for high-risk groups.

Moreover, the influenza virus has the intrinsic capacity to rapidly mutate over time, in a process called “antigenic drift” [5], and it may be affected by immune selective pressure. Thus influenza vaccines require updates each flu season and these updates need to be tailored differently for different countries. Due to the capacity of the influenza virus to elude the immune system, influenza vaccines require constant monitoring and must contain multiple virus strains. In fact, the influenza vaccine is typically composed of two IAV strains and one (trivalent vaccine) or two (quadrivalent vaccine) IBV strains [6].

In addition to antigenic drift, influenza viruses may also undergo the process of ‘antigenic shift’ in which a reassortment of genetic material of another virus family occurs creating a novel antigenic viral strain. The antigenic shift phenomenon may be responsible for a wide infection spreading leading to pandemics. In this way, throughout history, several influenza pandemics have occurred, such as the 1889 “Russian flu” (H2N2), the 1918–1920 “Spanish flu” (H1N1), the 1957–1958 “Asian flu” (H2N2), the 1968–1969 “Honk-Kong flu” (H3N2), and the 2009–2010 “Swine flu” (H1N1). These influenza pandemics have been responsible for several million infections and deaths worldwide.

Both the antigenic drift and antigenic shift processes underline the importance of developing large vaccination strategies to reduce the clinical impact of influenza infections and to prevent the spread of severe influenza pandemics worldwide. The tendency of influenza virus to accumulate mutations over time represents an important challenge for the vaccine industry that has to ensure the production of over 1.5 billion doses every year considering the existing technological approaches [7].

Currently, there are three different approaches to develop influenza vaccines: (i) conventional egg-based vaccines, (ii) cell-based vaccines, and (iii) the next-generation synthetic vaccines [8]. Then, licensed influenza vaccines can be based on live attenuated virus, inactivated virus, or recombinant hemagglutinin (HA) [9]. Live attenuated influenza virus vaccines are made of weakened viruses produced in a foreign host, such as live animals, animal cells, embryonated cells, and cultured cells [10]. Inactivated influenza virus vaccines consist of split viruses, produced via chemical disruption, or subunit antigens, HA and NA, extracted from the surface of the virus using chemical detergents [11]. Inactivated influenza vaccines are the most commonly used, because they are known to be safe and present low production costs.

During the last 50 years, the predominant method of producing vaccines has been to grow them in embryonated chicken eggs [12]. While it is a safe and proven process, this approach presents some significant negative aspects. The egg-based approach requires long production times, so that the process has to start several months before the start of the flu season. This can lead to mismatches between influenza vaccine strains and circulating strains [13]. Additionally, the egg-based manufacturing process may impact influenza vaccine effectiveness due to adaptation of the virus to growth in eggs: egg-adaptive mutations may occur and consequently alter viral antigenicity [14]. Egg-based vaccines also carry the risk of causing severe allergic responses to egg proteins in some patients. Non egg-based vaccine production could reduce some of these limitations lead to more effective influenza vaccines.

Two different non-egg based vaccines have been developed to address some of these limitations. The first, Flucelvax^®^ (Seqirus Inc., Holly Springs, NC, USA), approved in 2012 is a cell-based quadrivalent vaccine in which influenza virus is cultured in Madin-Darby Canine Kidneys (MDCK) cells [15]. The following year, in 2013, a new system of influenza vaccine production, the Flublock^®^ (Protein Sciences Corporation, Meriden, CT, USA), was approved. This system utilizes baculovirus to transfer the viral RNA using cloned HA genes [16]. Both Flucelvax^®^ and Flublock^®^ vaccines have the advantages of being non-egg based, but on the other hand, they are more expensive than the egg based ones. A recent study of real-world evidence has clearly shown that cell-based quadrivalent vaccines are more effective than egg-based quadrivalent vaccines in reducing in both hospitalization and health care costs attributed to influenza [14]. This improvement is likely to be due to the elimination of egg-adaptive mutations in the virus. Thus, cell-based approaches have the advantage not only of avoiding the egg-related limitations of allergic reactions and possible egg-shortages, but, more importantly, they reduce the risk of altered antigenicity due to the occurrence of mutations by virus egg adaptations, and this improves the effectiveness of the vaccine [17]. Even so, cell-based vaccines present significant disadvantages, such as the higher production costs and the problem of ensuring an adequate manufacturing capacity to serve the global demand for influenza vaccines. Yet, despite these limitations, the use of cell-based vaccines must be encouraged.

In 2016, another vaccine approach, the Fluad^®^ (Seqirus Inc., Holly Springs, NC 27540, USA), was developed. This is an egg-based system which utilizes an immunogenic cocktail, MF59, which is principally composed of squalene [18]. The MF59 enhances the immune response, recruiting immune cells to the site of inoculation. MF59-adjuvanted trivalent influenza vaccine (Fluad^®^) has been approved for elderly people >65 years, though it has been demonstrated that it may also be effective in children [6]. Recently, a test negative case-control study performed in Italy on elderly patients with severe acute respiratory infections (SARI) has shown that trivalent adjuvanted vaccine Fluad^®^ has a good effectiveness in preventing influenza, mainly against A(H1N1) strains rather than against B strains [19].

Moreover, real-life experience about the effectiveness of influenza vaccine is contrasting. A recent large test-negative controlled study performed in USA on about 15,000 patients has shown that inpatients and outpatients have to be considered as distinct populations and that influenza vaccination is effective in preventing influenza associated-hospitalizations [20]. Nevertheless, the results of a large observational study evaluating 170 million episodes of care in United Kingdom have been recently published and this study have revealed that the strategy to prioritize influenza vaccination in elderly people may be not as effective as believed in reducing influenza-related morbidity and mortality in this population [21]. However, large cohort studies comparing with each other the different influenza vaccines types effectiveness are still lacking; these studies might be very useful in evaluating the most appropriate vaccine approach in the different countries, in the different influenza seasons, and in the different age groups and comorbidities.

In the last few years, new approaches to developing and producing vaccines continue to be investigated. To be successful, next-generation vaccines must provide persistent and wide protection from the infection and, at the same time, their production should permit large-scale availability of the vaccine within a short time [22]. The next-generation seasonal influenza vaccines contemplate new strategies, such as egg-based, nanoparticle-based, peptide-based and nucleic acid-based approaches (Table 1). For example, Harding et al. have developed a strategy using engineered recombinant viruses grown in an egg-based culture which has the important advantage of avoiding the common egg-adaptive mutations of other egg-based systems [23]. Another approach, similar to the baculovirus-based vaccine Flublock^®^, is a nanoparticle-based approach utilizing nanoparticles as vectors to assemble HA into a vaccine. This allows the inclusion of a much larger amount of HA and thus a stronger immune response, while also avoiding the egg-related disadvantages [24]. Furthermore, peptide-based vaccines rely on the synthesis of specific purified influenza epitopes using liposomes or virosomes as adjuvant and delivery mechanism for the antigen [25]; they avoid egg-related limitations and are able to provide broad protection by strongly activating the immune system. However, they utilize a complex manufacturing system with high costs. Finally, nucleic acid-based vaccines use recombinant DNA/RNA molecules of the specific viral antigen that are inoculated into a plasmid and amplified using bacteria, such as *Escherichia coli* [26]. This technique is simpler to achieve but may have a limited efficacy due to an activation of innate immune response.

Among new vaccine approaches, adenoviral vector-based vaccine platform is currently being investigating. Vector-based vaccines are able to elicit an immunogen-specific innate and adaptive immune response through both Toll-like receptor (TLR)-dependent and TLR-independent pathways without the need for an adjuvant. Adenoviral vector-based vaccines are egg-independent and can be produced on a large scale within a short time [22]. Adenoviral vector-based vaccines are currently being demonstrating effective in several pre-clinical and phase II clinical trials, but further studies are needed to confirm these results and evaluate if these vaccines may be used in the different susceptible groups. 

Furthermore, there are several host-associated factors that can modify the efficacy of influenza vaccination, such as age, race, gender, obesity, gut microbiota composition, and hormonal variability between the sexes and during pregnancy [27]. Compounding matters is the phenomenon of immune-senescence which causes impaired innate and adaptive immune responses typical in the elderly. At the same time, the age-related gut microbiota composition variability can further influence this process and modify the effectiveness of vaccination. Gut microbiota composition can also vary according to gender, race, body composition, diet, hormonal changes, drug consumption, and several human chronic pathologies [17,28,29]. These factors can heavily influence the efficacy of influenza vaccines and may explain the wide inter-individual variability.

The efficacy of current available influenza vaccines can vary among seasons from 10 to 65% [30]. This broad variability of efficacy is due to several factors, not only the intrinsic yearly virus strain mutation tendency (which is a consequence of antigenic drift), but also the vaccine-manufacture process, and host-related factors (Figure 1). Ideally, to achieve universal influenza prevention, a vaccine would need to be highly effective in all age groups, inexpensive, and safe, and able to provide persistent and extensive immune-protection. Additionally, the vaccine should be potentially available globally without limitations for age, sex, and clinical comorbidities. 

To date, a significant problem of vaccines’ effectiveness is that their specificity is inversely correlated to their universality. To improve the universality of vaccine effectiveness, subtype-specific, multi-subtype, and pan-group vaccines need to be developed [11]. At present, we are far from having a universal flu A and B vaccine, and the best objective is to obtain an all strains-specific influenza vaccine. The goal is to produce an influenza vaccine that meets two important criteria: durable protective immunity and universal protection producing a cross-reactive immune response to the different influenza virus strains [31].

Today the importance of having an effective seasonal influenza vaccine is more critical than ever, considering the recent Coronavirus disease-19 (COVID-19) pandemic that, so far, has caused millions of infections and several hundreds of thousands deaths worldwide. A universal vaccination strategy against influenza will facilitate herd immunity among the world population and thus protect people in whom the vaccine is not effective [32]. However, it is important consider that the interventions adopted to limit and prevent the spread of COVID-19 will also limit the spread of influenza: since both viruses are mainly transmitted by respiratory droplets, social distancing, the use of face masks, quarantine, isolation, and the variable lockdown measures will be also contribute in decreasing influenza incidence [33]. Furthermore, universal influenza vaccination, especially next winter in which seasonal influenza virus will circulate in parallel with SARS-CoV-2 virus, will permit doctors to better discriminate between these two infections and thus relieve the influenza-related hospitalization to better cure COVID-19 syndrome patients.

## Figures and Tables

**Figure 1:**
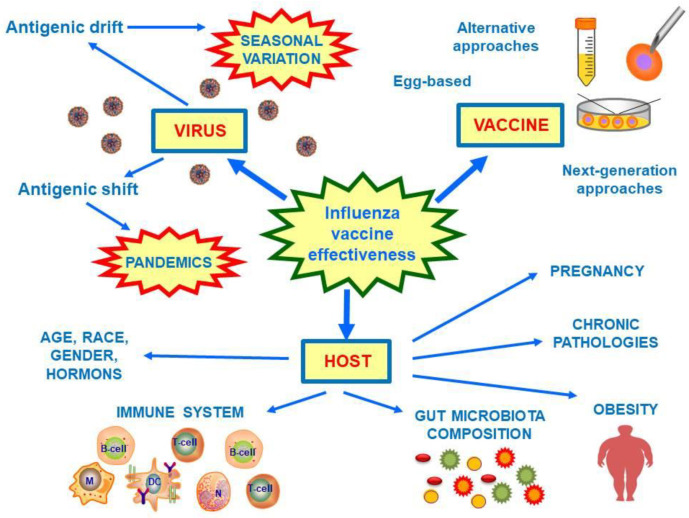
Factors involved in the influenza vaccine effectiveness. Influenza vaccine effectiveness is modified by virus mutation tendency, vaccine manufacture process, and host factors. Influenza viruses are characterized by seasonal antigenic mutations (antigenic drift), and they may undergo to periodical antigenic shift responsible for pandemics. To date, there several influenza vaccine types; the most common used are egg-based vaccine, but alternative approaches are available and next-generation ones are developing. Finally, several host factors may further influence vaccine effectiveness, such as age, race, gender, hormones, pregnancy, obesity, chronic pathologies, and gut microbiota composition. Abbreviations: M, macrophage; DC, dendritic cell; N, neutrophil.

**Table 1:** Advantages and disadvantages of different influenza vaccines.

Type of Vaccine	Advantages	Disadvantages	Reference
**current approach**			
Egg-based vaccine	- Low cost - Safe and proven process	- Long production time - Viruses may grow poorly in chicken eggs - Egg shortages - Severe allergic responses to egg proteins in some patients	[12]
**approved alternative approaches**			
cell-based vaccine (*Flucelvax^®^)*	- Avoid egg-related limitations	- High costs - Complex manufacturing process	[15]
baculovirus-based vaccine (*Flublock^®^)*	- Avoid egg-related limitations	- High costs - Complex manufacturing process	[16]
adjuvanted egg-based vaccine (*Fluad^®^)*	- Adjuvanted - Easy manufacturing process	- Egg-adaptation - Severe allergic responses to egg proteins in some patients - Only approved for elderly people >65 years	[18]
**next-generation approaches**			
engineering virus/egg-based approach	- Avoid the common egg-adaptive mutations of other egg-based systems - Low cost	- Egg shortages - Severe allergic responses to egg proteins in some patients	[23]
nanoparticle-based approach	- No virus use - Avoid egg-related limitations - Allows the inclusion of a larger amount of HA and thus a stronger immune response	- Complex manufacturing process - Uncertain timelines	[24]
peptide-based approach	- No virus use - Avoid egg-related limitations - Provide broad protection by strongly activating the immune system	- High costs - Complex manufacturing process	[25]
acid nucleic-based approach	- No virus use - Easy manufacturing process	- Limited efficacy due to an activation of innate immune response	[26]
